# The role of bacteria in lactational mastitis and some considerations of the use of antibiotic treatment

**DOI:** 10.1186/1746-4358-3-6

**Published:** 2008-04-07

**Authors:** Linda J Kvist, Bodil Wilde Larsson, Marie Louise Hall-Lord, Anita Steen, Claes Schalén

**Affiliations:** 1 Department of Obstetrics and Gynaecology, Helsingborg Hospital, 251 87, Sweden; 2 Department of Nursing, Karlstad University, 651 88, Sweden; 3 Department of Nursing, Gjovik University College, Norway; 4 Department of Clinical Microbiology and Immunology, University Hospital, Lund, Sweden

## Abstract

**Background:**

The role of bacterial pathogens in lactational mastitis remains unclear. The objective of this study was to compare bacterial species in breast milk of women with mastitis and of healthy breast milk donors and to evaluate the use of antibiotic therapy, the symptoms of mastitis, number of health care contacts, occurrence of breast abscess, damaged nipples and recurrent symptoms in relation to bacterial counts.

**Methods:**

In this descriptive study, breast milk from 192 women with mastitis (referred to as cases) and 466 breast milk donors (referred to as controls) was examined bacteriologically and compared using analytical statistics. Statistical analyses were also carried out to test for relationships between bacteriological content and clinical symptoms as measured on scales, prescription of antibiotics, the number of care contacts, occurrence of breast abscess and recurring symptoms.

**Results:**

Five main bacterial species were found in both cases and controls: coagulase negative staphylococci (CNS), viridans streptococci, *Staphylococcus aureus *(*S. aureus*), Group B streptococci (GBS) and *Enterococcus faecalis*. More women with mastitis had *S. aureus *and GBS in their breast milk than those without symptoms, although 31% of healthy women harboured *S. aureus *and 10% had GBS. There were no significant correlations between bacterial counts and the symptoms of mastitis as measured on scales. There were no differences in bacterial counts between those prescribed and not prescribed antibiotics or those with and without breast abscess. GBS in breast milk was associated with increased health care contacts (p = 0.02). Women with ≥ 10^7 ^cfu/L CNS or viridans streptococci in their breast milk had increased odds for damaged nipples (p = 0.003).

**Conclusion:**

Many healthy breastfeeding women have potentially pathogenic bacteria in their breast milk. Increasing bacterial counts did not affect the clinical manifestation of mastitis; thus bacterial counts in breast milk may be of limited value in the decision to treat with antibiotics as results from bacterial culture of breast milk may be difficult to interpret. These results suggest that the division of mastitis into infective or non-infective forms may not be practically feasible. Daily follow-up to measure the subsidence of symptoms can help detect those in need of antibiotics.

## Background

Appropriate treatment for inflammatory symptoms of the breast in lactating women has been under discussion in the scientific literature for some time. One reason why consensus has not been reached is that the clinical spectrum of "mastitis" covers a range from focal inflammation with minimal systemic response to septicaemia [1]. According to a review by the World Health Organization (WHO), the incidence of mastitis varies greatly, from 2.6% to 33%, among breastfeeding women [2]. This suggests that due to difficulties in the definition of the term "mastitis" [3], researchers might not have been investigating comparable groups of women. Furthermore, the lack of internationally agreed scales for the measurement of symptoms creates difficulties in the use of meta-analyses.

The role of bacterial pathogens in lactational mastitis is unclear [1-4]. In the 1980s, Thomsen et al suggested that levels > 10^6 ^cfu/L of pathogenic bacteria in breast milk was an indication for antibiotic treatment since this level, together with leukocytosis was indicative of infection [5,6]. Others have suggested that untreated cases may recover as quickly as treated cases [7] and that bacteriological examination of breast milk may be of limited value [8].

The aims of this study were to compare bacterial species in breast milk of mothers with mastitis and of healthy breast milk donors and to evaluate in relation to bacterial counts, the use of antibiotic therapy, the symptoms of mastitis as measured on scales, number of health care contacts, occurrence of breast abscess, occurrence of damaged nipples and recurrence of symptoms.

## Methods

### Study population

The case group consisted of 205 women who contacted a breastfeeding clinic in southern Sweden during 2002 – 2004 because of inflammatory symptoms of the breast during lactation and had agreed to join a randomised controlled trial of care interventions [4]; registration number of the RCT is: NCT00405158. The incidence of mastitis in the uptake area was estimated as 6% of the breastfeeding population [4]. Of the 205 women, 192 (94%) had their breast milk sampled and sent for bacteriological investigation. A follow-up questionnaire inquiring about recurrent symptoms and the women's views on care given was sent by post to the cases, six-weeks following their last contact with the breastfeeding clinic. A total of 176 (84%) returned the questionnaire.

The control group consisted of 466 healthy, prospective breast milk donors living in the same geographical area as the case group and studied during the same period of time. According to Swedish recommendations, women with an established lactation who wish to donate breast milk for use in neonatal units are obliged to leave milk for bacterial analysis before being accepted as donors.

### Ethical considerations

Ethical approval for the randomised controlled trial (RCT) was granted by the Committee for Medical Research Ethics, Lund University Hospital, Sweden (protocol number LU 592–00). Two of the authors (CS and AS) are employed at the laboratory where the specimens are tested and therefore had access to the material. After analysis of milk samples and reporting of results to the neonatal units who submitted the samples, a database, without any means of identification of individual samples was set up. Since personal data of the controls were unknown to the researchers, results of their breast milk cultures could not be traced to individuals.

### Signs and symptoms of breast inflammation

Women were considered to have mastitis when at least two of the following signs or symptoms were present: breast erythema, increased breast tension not relieved by breastfeeding, maternal fever, pain in the breast and lumps in the breast tissue [4]. A midwife at the breastfeeding clinic carried out the initial measurement of signs and symptoms together with the woman. Scores for these signs and symptoms were estimated on scales, which were tested for internal consistency using Cronbach's alpha [9]. The alpha score on contact day three was 0.79 [4,10]. Scores were added to create a Severity Index (SI), range 0 (least severe) to 19. Table 1 shows the values for the scales. At subsequent daily telephone follow-ups, the women were asked to report their scores in relation to their scores on the previous day. The same midwife carried out the telephone follow-ups as far as possible but this was not always the case. The number of midwives involved in the study was seven.

**Table 1:** The scales used for the measurements of mastitis signs and symptoms in the RCT

**Erythema**	**Breast tension**
No redness = 0	No change = 0
Slight redness in limited area = 1	Firm, no tenderness = 1
Redness in limited area = 2	Tense, not uncomfortable = 2
Bright red in limited area = 3	Tense and uncomfortable = 3
Bright red over most of the breast = 4	Tense and painful = 4
	Very tense and very painful = 5

Pain was measured by Visual Analogue Scale: 0 = no pain, 10 = worst possible pain. Severity Index = Erythema + Breast tension + Pain: range 0–19

### Collection of samples

Both cases and controls were asked to provide a sample of breast milk for bacterial culture and the samples were all examined at the same laboratory. The nipple and areola of the affected breast was cleaned with normal saline solution; the woman was given non-sterile surgical gloves and asked to manually express several drops of milk, which were discarded before collecting the specimen directly into the test tube. In cases of bilateral symptoms (14%), milk was sampled from the breast which was most affected. The controls provided a sample of milk pooled from both breasts. In the case group, the women were informed that the results of culture would be available after two to three days, by which time it was expected that they would be on the way to recovery. A sample of breast milk was obtained from each of the 192 cases and all 466 of the control subjects.

### Bacteriological analysis of breast milk

Breast milk samples were refrigerated until transport to the laboratory within 24 hours. Tenfold dilutions of milk were plated on horse blood agar (HBA) and incubated at 37°C aerobically for two days. Bacterial species were quantified and identified according to standard methods.

### Statistical analysis

The material was analysed using SPSS version 14.0 (SPSS, Inc., Chicago, USA). Five main bacterial species occurred in sufficient numbers of specimens to allow statistical comparisons between cases and controls. Odds Ratios (OR) with 95% confidence intervals (CI) were calculated for the occurrence of mastitis for each of these species. Pearson's correlation test was used to assess possible correlations between bacterial counts and symptoms at first contact: maternal temperature, breast erythema, increased breast tension, pain and SI (0–19). Differences in mean bacterial counts between cases who received or did not receive antibiotics and between those with and without abscess were assessed using the independent samples t-test. In accordance with the level of bacterial content described by Thomsen et al [5,6] variables for the five main bacterial species in the mastitis group were dichotomised to ≤10^6 ^cfu/L milk and ≥10^7 ^cfu/L milk. Odds Ratios with 95% confidence intervals were then calculated for the proportions of cases with higher or lower levels of the five bacterial categories *vs*. prescription of antibiotics, occurrence of residual symptoms within six weeks and for the occurrence of damaged nipples. The independent samples t-test was used to assess whether those with any one of the five main bacterial species in breast milk required prolonged contact with the breastfeeding clinic. Significance was assumed at the 0.05 level.

## Results

### Comparison of bacterial findings in cases and controls

Microbiological findings are summarized in Table 2. The five main bacterial species found in cases and controls were coagulase negative staphylococci (CNS), viridans streptococci, *Staphylococcus aureus *(*S. aureus*), Group B streptococci (GBS) and *Enterococcus faecalis*. CNS were detected significantly more often in the milk of the healthy controls than among cases (OR: 0.60, 95%CI: 0.35, 0.91). In contrast, three of the other species were present significantly more often among cases than controls: viridans streptococci: (OR: 1.43, 95%CI: 1.02, 2.01) *S. aureus*: (OR: 1.81, 95%CI: 1.29, 2.60) and GBS: (OR: 2.40, 95%CI: 1.50, 3.71).

**Table 2:** Bacterial findings in breast milk of women with and without symptoms of mastitis: Odds Ratios with 95% confidence intervals for symptoms of mastitis

	**Healthy donors (*n *= 466)**		**Women with mastitis (*n *= 192)**		
**Bacterial Species**	**Occurrence of bacteria in breast milk n (%)**	**Mean (SD) counts (cfu/L)**	**Occurrence of bacteria in breast milk n (%)**	**Mean (SD) counts (cfu/L)**	**Odds Ratio for occurrence of mastitis symptoms (95%CI)**
**CNS**	419 (90%)	10^6(± 1.1)^	160 (83%)	10^6(± 1.1)^	0.60 (0.35, 0.91) p = 0.02
**Viridans Streptococci**	233 (50%)	10^5(± 1.0)^	113 (59%)	10^5(± 1.0)^	1.43 (1.02, 2.01) p = 0.04
***Staphylococcus aureus***	145 (31%)	10^6(± 1.2)^	87 (45%)	10^6(± 1.3)^	1.81 (1.29, 2.60) p = 0.001
**Group B streptococci**	47 (10%)	10^6(± 1.5)^	41 (21%)	10^6(± 1.4)^	2.40 (1.50, 3.71) p = <0.001
***Entercoccus faecalis***	28 (6%)	10^6(± 1.1)^	16 (8%)	10^6(± 1.1)^	0.70 (0.36, 1.43) p = 0.36
**Group G streptococci**	6 (1%)	10^6^	3 (2%)	10^7^	
**Group A streptococci**	3 (1%)	10^7^	2 (1%)	10^7^	
**Pneumococci**	4 (1%)	10^7^	5 (3%)	10^5^	
**Corynebacteria**	2 (0.4%)	10^4^	1 (0.5%)	10^5^	
**Enterobacteriaceae**	36 (8%)	10^6^	2 (1%)	10^6^	
**Acinetobacter species**	12 (3%)	10^6^	0	0	
**Pseudomonas species**	24 (5%)	10^7^	1 (0.5%)	10^6^	
**Bacillus species**	5 (1%)	10^5^	1 (0.5%)	10^4^	
**Propionibacterium species**	3 (1%)	10^6^	2 (1%)	10^6^	
**Candida species**	4 (1%)	10^5^	4 (2%)	10^5^	

CNS = coagulase negative staphylococci, SD = Standard Deviation

### Correlations between symptoms and bacterial counts

There were no significant correlations between bacterial counts of the five main species in the breast milk and maternal temperature, breast erythema, breast tension, pain, or the Severity Index at first contact (Table 3).

**Table 3:** Correlations between symptoms and bacterial counts in women with mastitis (n = 192)

**Bacterial species**					
	CNS	Viridans streptococci	*Staphylococcus aureus*	Group B Streptococci	*Enterococcus faecalis*
**Symptoms**					
Fever	r = 0.20 p = 0.06	r = 0.14 p = 0.21	r = 0.05 p = 0.72	r = 0.09 p = 0.63	r = 0.15 p = 0.62
Erythema	r = 0.06 p = 0.44	r = 0.008 p = 0.93	r = 0.15 p = 0.16	r = -0.21 p = 0.30	r = 0.24 p = 0.38
Unrelieved breast tension	r = -0.04 p = 0.64	r = 0.11 p = 0.30	r = -0.01 p = 0.95	r = -0.05 p = 0.75	r = 0.41 p = 0.11
Pain	r = -0.02 p = 0.90	r = 0.74 p = 0.43	r = 0.05 p = 0.62	r = -0.24 p = 0.14	r = 0.42 p = 0.90
Severity Index	r = -0.01 p = 0.91	r = 0.07 p = 0.50	r = 0.09 p = 0.45	r = -0.21 p = 0.18	r = 0.31 p = 0.30

*r *= Pearson's correlation coefficient, CNS = coagulase negative staphylococci

### Bacteria and number of contact days with breastfeeding clinic

Occurrence of GBS in the breast milk was associated with a significant increase in the number of contact days with the breastfeeding clinic (t = -2.44, p = 0.02). In contrast, presence of CNS, viridans streptococci or *S. aureus *did not affect the number of health care contact days.

### Antibiotic therapy, breast abscess, recurrence of symptoms and damaged nipples in relation to bacterial counts

Seven of the women (3.3% of the cases) were prescribed antibiotics on the basis of their symptoms alone, before results of bacterial cultures were available. A total of 24 (11.4%) cases were prescribed antibiotics on the basis of culture results, including those who developed a breast abscess during contact with the breastfeeding clinic. Thus, a total of 31 (15%) women in the case group received antibiotic treatment. Table 4 shows the outcome of culture from the breast milk of women who were prescribed antibiotics on the basis of symptoms alone and women who developed a breast abscess during contact with the breastfeeding clinic. Among the cases there were no significant differences in bacterial counts of those given (n = 31) *vs *not given (n = 161) antibiotics or those with (n = 7) *vs *without a breast abscess (n = 185). Less than half (42%) of the 31 women who were given antibiotics had *S. aureus *in their breast milk. The odds for damaged nipples was increased when ≥ 10^7 ^cfu/L of CNS or viridans streptococci was present in the breast milk: CNS: OR: 2.51 (95%CI: 1.33, 4.61) and viridans streptococci: OR: 2.54 (95%CI: 1.34, 4.90).

**Table 4:** Bacterial findings in breast milk of i) women prescribed antibiotics on the basis of symptoms alone and ii) women who presented with or developed abscess during treatment

	**Concentrations of bacterial species expressed in cfu/L**					
	**CNS**	***S. aureus***	**Viridans streptococci**	***Pneumococci***	**Group B streptococci**	**Group A streptococi**
Women prescribed antibiotics on the basis of their symptoms						
No.1	10^6^				10^8^	
No.2	10^8^	10^5^				
No.3	10^6^		10^5^			
No.4		10^4^	10^5^			
No.5	10^8^	10^8^				
No.6		Not quantified				Not quantified
No.7 No culture carried out						
						
Women who presented with or developed breast abscess during treatment						
No.1	10^7^					
No.2	10^6^	10^7^	10^7^			
No.3	10^7^	10^7^				
No.4	10^6^	10^4^				
No.5	10^6^	10^7^	10^5^			
No.6	10^6^			10^6^		
No.7	10^6^	10^6^				

At a six-week postal follow-up of the cases, 21 women (12% of respondents) reported contacting health care providers because of recurrent symptoms and antibiotics had been prescribed for eight of them. Out of these eight women, five had previously received a course of antibiotic treatment for their initial symptoms. One further woman reported a breast abscess. There were no reports of infected infants in the case group. Of the 21 who contacted health services because of recurrent symptoms within six weeks, ten were positive for *S. aureus*. There were no increased odds for recurrence of symptoms when the bacterial counts were ≥10^7 ^cfu/L for any of the five species.

## Discussion

It was shown, somewhat surprisingly, that many women with potential pathogens in their breast milk, even at high bacterial counts, either recovered spontaneously from mastitis or did not have any symptoms of mastitis (as seen among control women). Furthermore, it was demonstrated that increasing bacterial counts did not influence the clinical manifestation of mastitis. The breast milk of women who were given antibiotics because of their symptoms, did not contain higher bacterial counts than those who were not given antibiotics. These results and the finding that so many healthy women harboured potential pathogens in their breast milk may indicate that the division of mastitis into infective or non-infective forms may not be practically feasible. Although only 15% of women with mastitis were given antibiotics, most of them recovered spontaneously with few cases of recurrent symptoms within 6 weeks requiring antibiotic treatment.

The diagnostic criteria for mastitis used in this study consisted of clinical signs and symptoms, which are in the main subjective. This implies that the scope of illness reported in the case group may have been quite broad, ranging from mild to very severe. However, the 6% incidence of mastitis in the breastfeeding population was low compared to the range reported by WHO (2.6% – 33%), which suggests that the women in the RCT were correctly diagnosed. The incidence of 3.3% of breast abscess development in the group with mastitis can be compared to data from a recent study from Australia, which showed that 14.5% of breastfeeding women were treated with antibiotics and 2.9% of the women with mastitis developed an abscess [11]. Leukocyte counts have previously been used as a marker of infective mastitis [5]. However, it was not possible in the present study to measure leukocyte levels since the cases were originally included in a RCT of care interventions where measurement of infection was not the primary aim. Bacterial content in breast milk may vary over time and by geographical location and therefore the findings of this study may not be applicable to all communities.

Our analysis suggested that the two most abundant bacterial species or groups found, CNS and viridans streptococci, were not linked to clinical mastitis; these are well established as major components of the resident skin or throat flora and possibly of importance for protection against pathogens [12]. Interestingly, Heikkilä and Saris found that human breast milk contains commensal bacteria that inhibit *S. aureus *and may prevent maternal breast infections [13]. Two potentially pathogenic species, *S. aureus *and GBS, were recovered more frequently in mastitis cases as compared to controls, indicating an active role in mastitis. In particular, *S. aureus *has earlier been reported as a primary causative organism in mastitis [14-16] and might conceivably be expected to give rise to breast abscess. The breast milk samples of five of the seven women with a breast abscess was positive for *S. aureus*, but these figures are too small to allow any conclusions to be drawn. The inflammation in mastitis occurs in the connective tissue of the breast and for this reason causative organisms may be difficult to isolate from breast milk.

It was stated in a WHO review on mastitis that antibiotic treatment is indicated if either cell or bacterial colony counts are available and indicate infection or symptoms are severe from the beginning or a nipple fissure is visible or symptoms do not improve after 12–24 hours of improved milk removal [2]. If these criteria had been followed, many more than the 15% of cases, as reported here, would have received antibiotics. Reports from industrialised countries such as Australia [17-19] and USA [20,21] have shown that 77% – 97% of women with lactational mastitis are prescribed antibiotics. The findings of our study do not appear to support this extensive use of antibiotic treatment for mastitis during lactation. It is important for all communities to avoid imprudent use of antibiotics because of the spread of methicillin resistant *S. aureus *(MRSA) and other multi-resistant pathogens. It has also been considered that maternal antibiotic therapy might disturb the commensal flora of the upper airways in breastfed infants [22].

The major rationale for treatment of mastitis by antibiotics is to avoid the development of breast abscess. However, empirical evidence for a continuum from mastitis to abscess is not readily available and the WHO review suggests that breast abscess may occur seemingly spontaneously, irrespective of previous mastitis [2]. Nonetheless, lactational mastitis has the potential for serious sequelae such as breast abscess and in rare cases septicaemia [1] and if clinical signs and symptoms and bacterial culture of breast milk are unhelpful, we need to consider how to aid in the decision to prescribe antibiotics. In order to select which women will not recover without antibiotic treatment, it is necessary to maintain daily contacts with the women to assure that symptoms are subsiding. Although, as reported earlier [4] the mean number of contact days with a breastfeeding clinic was 5 (± 2.9), it is important, in our opinion, that symptoms improve within 24 to 48 hours after initiation of care interventions. Scales to measure symptoms may assist clinicians in judging whether improvement has occurred. Continuity of care provider will also ensure that women are adequately treated. Those not improving will need a new consultation with a view to antibiotic treatment. At this time, results of bacterial culture may be of value for choice of antibiotic.

The frequent occurrence of GBS in both cases and healthy mothers was notable. GBS is a leading cause of serious infection in neonates [23] but no such infections were reported in the context of this study. Whether certain components of breast milk may protect infants and mothers from invasive GBS infection will be an important area for future research. Another avenue for investigation might be to consider why many women who harbour *S. aureus *do not develop mastitis. Knowledge of how often breast abscess occurs during lactation without any noticeable episode of preceding breast inflammation might also give us more understanding of the process. Well-designed RCTs of antibiotic therapy for treatment of lactational mastitis would help define the optimal level of such treatment for this group. New research could also include measurement of leukocyte levels.

## Conclusion

Potentially pathogenic bacteria, often at high levels, were found in breast milk from both healthy donors and women with lactational mastitis. Increasing bacterial counts did not affect the clinical manifestation of mastitis, thus bacterial counts in breast milk may be of limited value in the decision to treat with antibiotics and results from bacterial culture of breast milk may be difficult to interpret. These results suggest that division of mastitis into infective and non-infective forms may not be practically feasible. Daily follow-up to measure the subsidence of symptoms can help identify those in need of antibiotic treatment.

## Competing interests

The author(s) declare that they have no competing interests.

## Authors' contributions

LJK, MLH-L, BWL and CS designed the study. AS and CS provided expert advice on collection of samples and interpretation of microbiological findings. Statistical analyses were carried out by LJK who drafted the manuscript. The manuscript was modified after discussions with MLH-L, BWL and CS.
